# *Exploring PIEZO1* DNA methylation in infants with neurodevelopmental disorders

**DOI:** 10.3389/fpsyg.2025.1593609

**Published:** 2025-07-31

**Authors:** Eleonora Mascheroni, Fabiana Mambretti, Laura Cordolcini, Annalisa Castagna, Elisa Rosa, Niccolò Butti, Andrea Citterio, Nivedita Agarwal, Rosario Montirosso

**Affiliations:** ^1^Scientific Institute, IRCCS E. Medea, 0-3 Center for the at-Risk Infant, Bosisio Parini, Italy; ^2^Scientific Institute, IRCCS E. Medea, Molecular Biology Lab, Bosisio Parini, Italy; ^3^Scientific Institute, IRCCS E. Medea, Diagnostic Imaging and Neuroradiology Unit, Bosisio Parini, Italy

**Keywords:** neurodevelopmental disorders, epigenetics, DNA methylation, Piezo1, *PIEZO1*

## Abstract

**Introduction:**

Neurodevelopmental disorders (NDs) are a range of heterogeneous clinical conditions associated with dysfunctional brain development. Variations in DNA methylation (DNAm) have been reported in patients with NDs. Piezo1, which is encoded by the *PIEZO1* gene, is a mechanosensitive ion channel protein involved in mechanotransduction across many physiological systems. Its regulation is involved in several diseases of the Central Nervous System (CNS) during adulthood and aging. Although *PIEZO1* gene expression is susceptible to epigenetic regulation associated with pathological phenotypes during development, no previous study has explored *PIEZO1* DNAm in infants with NDs.

**Methods:**

*PIEZO1* methylation in 15 CpG sites was assessed in 24 infants with NDs and in 22 infants with typical development (TD) aged between 3 and 36 months.

**Results:**

A principal component analysis (PCA) was run and yielded two factors: principal component1 (PC1) comprising 7 CpG sites and principal component2 (PC2) comprising 8 CpG sites. In PC2, DNAm levels were lower in infants with NDs compared to TD, suggesting hypomethylation in the clinical group, which, in turn, might impact the degree of Piezo1 protein expression.

**Conclusion:**

We speculate that *PIEZO1* hypomethylation as a potential epigenetic mark could contribute to the poorer mechanical properties of brain tissue in infants with NDs by altering the Piezo1 expression patterns. These findings suggest that the *PIEZO1* DNAm status could serve as an early epigenetic marker of NDs, offering promising implications for identifying underlying mechanisms involved in their onset.

## Introduction

### Background

Neurodevelopmental disorders (NDs) are a range of heterogeneous clinical conditions such as cerebral palsy, developmental delay, intellectual disability, language delay, and epilepsy. Their pathogenesis is due to an altered morphogenesis and/or histogenesis (Morris et al., 2013). This results in dysfunctional brain development due to alterations in several biochemical mechanisms during neurodevelopment, so that an alteration of several biochemical mechanisms during neurodevelopment results in a dysfunctional brain development, even when it is not detectable by neuroimaging techniques (Momen et al., 2011). Although NDs are associated with dysfunctional brain development (Hemed and Melosh, 2023), only 30.7% of patients exhibit brain abnormalities at the MRI scan (Engbers et al., 2010), thus making it necessary to use other diagnostic methods such as cytogenetic analyses, metabolic screening, and EEG to indirectly identify potential brain impairments (Williams, 2004).

Recent research indicates that the mechanical properties of the cell microenvironment can regulate physiological processes (Koser et al., 2016) and contribute to the progression of brain dysfunction (Zheng et al., 2023). Piezo1 is a transmembrane protein identified as a mechanosensitive receptor expressed in sensory and non-sensory neurons. It responds to mechanical stimulation by converting it into electrical and chemical signals within the cells (Coste et al., 2010; Coste et al., 2012). The process of mechanotransduction mediated by Piezo1 is crucial for maintaining cellular function. A dysregulated process is thought to be involved in the onset of a wide range of diseases (Liu et al., 2022). According to a recent review, the activity of the mechanosensitive Piezo1 channel is associated with health and disease of the Central Nervous System (CNS; Zong et al., 2023) and its regulatory role is apparently critical not only during adulthood and aging but also during neurodevelopment (Chi et al., 2022).

Epigenetic mechanisms, such as DNA methylation (DNAm), play an important role in the regulation of many physiological processes, including CNS functioning (Feng and Fan, 2009). Variations in DNAm have been reported in patients with NDs (Sadikovic et al., 2019) and several NDs have been associated to alterations of genes encoding proteins that are part of the epigenetic methylation machinery. A number of consequences are expected to result from the malfunctioning of these proteins, including an impact on the development of the nervous system (Mastrototaro et al., 2024). Although very little is known about the epigenetic modifications of the *PIEZO1* gene (also known as *FAM38a*), which encodes the Piezo1 protein (Zarychanski et al., 2012), few studies have documented methylation changes associated with pathological phenotypes during development, such as the Opioid Withdrawal Syndrome in neonates (Radhakrishna et al., 2021) and obesity in childhood (Ren et al., 2024). In the current study, we hypothesized that epigenetic modifications of *PIEZO1* might impact the Piezo1 protein expression and contribute to modifying its regulatory role also in the neurodevelopmental processes. To investigate whether potential epigenetic modifications of *PIEZO1* could be associated with early brain dysfunction, we analysed *PIEZO1* DNAm in infants with a diagnosis of ND aged between 3 and 36 months.

### Mechanical forces, Piezo1 and brain functioning

Although the influence of genetic and chemical factors on neural development has been widely investigated, recent studies have revealed the significant impact of mechanical forces on neural development and function (Abuwarda and Pathak, 2020). A stable mechanical environment is essential for proper brain development. Key stimuli during early life, such as tissue stiffness, fluid shear flow, and hydrostatic forces from cerebrospinal fluid, contribute to normal neurophysiological processes (Barnes et al., 2017; Javier-Torrent et al., 2021). However, the molecular mechanisms responsible for sensing and transducing these mechanical signals within the developing brain remain poorly understood.

Mechanosensitive ion channels have recently gained attention as important mediators of mechanotransduction, converting physical forces into biochemical signals that regulate critical cellular functions such as growth, migration, adhesion, morphogenesis, gene expression, and fluid homeostasis (Coutino and Mayor, 2021; Martino et al., 2018; Gu and Gu, 2014). Among these, four different families have been classified in mammals: epithelial sodium channel/degenerin (ENaC/DEG), transient receptor potential channel (TRP), two-pore domain potassium channel (K2P), and PIEZO channels (Prevarskaya et al., 2018). Evidence suggests that mutations in genes encoding brain ion channels from ENaC/DEG and K2P ion channel families are linked to NDs, emphasizing the broader role of mechanotransduction in brain dysfunction (D'Adamo et al., 2020).

Piezo1 has recently emerged as a particularly important mechanosensitive ion channel. Encoded by the PIEZO1 gene, Piezo1 is a nonselective Ca2 + channel that is highly conserved across species and expressed in several tissues of organisms, proving to be biologically important (Otero-Sobrino et al., 2023). In the CNS, Piezo1 is crucial for mediating various neurophysiological processes, such as neuronal growth and development, axon extension, glial cell migration, regulation of glial cell responsiveness, and activation of immune cells (Zheng et al., 2023). Moreover, Piezo1 is essential for the development of meningeal lymphatic vessels, which play a critical role in maintaining brain homeostasis by facilitating immune responses, removing waste, and regulating cerebrospinal fluid dynamics (Antila et al., 2017; Choi et al., 2024).

The regulation of *PIEZO1* gene expression may be crucial for some CNS diseases (Zheng et al., 2023). Recent studies suggest that PIEZO1 expression is epigenetically regulated through DNA methylation (DNAm), particularly in disease contexts. For example, in gliomas, higher PIEZO1 expression—associated with lower DNAm levels—correlates with tumour aggressiveness and a stiffer mechanical microenvironment, further aggravating disease progression (Chen et al., 2018; Zhou et al., 2020). Similarly, it was observed that *PIEZO1* expression is associated with Alzheimer’s disease pathogenesis and progression in the cerebellum (*in vitro* cerebellar cells) through DNAm (Li et al., 2022; Velasco-Estevez et al., 2018). Thus, the modulation of *PIEZO1* gene expression through DNAm modifications may regulate the mechanical environment of the brain.

### The current study

Although most evidence on *PIEZO1* and its epigenetic regulation comes from adult CNS studies, emerging findings suggest it is relevant during early brain development. The CNS undergoes rapid maturation in infancy—a period highly sensitive to genetic and epigenetic influences (Silbereis et al., 2016). Mechanical forces have been identified as key regulators of early neural development (Otero-Sobrino et al., 2023). Given Piezo1’s role in mechanotransduction and its known epigenetic modulation in adult CNS disorders, alterations in PIEZO1 DNAm during infancy may similarly affect neurodevelopmental trajectories.

In light of this, the present study analysed the epigenetic status of the Piezo-correlated gene (i.e., *PIEZO1* DNAm) like a proxy measure of the Piezo1-protein expression. We investigated *PIEZO1* DNAm status in infants with NDs and in infants with typical development (TD). We could not formulate strong hypotheses because no prior research had directly assessed the association between NDs and PIEZO1 methylation in infancy. However, considering that *PIEZO1* DNA hypomethylation has been associated with dysfunctional brain functioning, such as in Alzheimer’s disease (Li et al., 2022; Velasco-Estevez et al., 2018), we expected that infants with NDs would exhibit lower PIEZO1 DNAm levels compared to infants with TD.

## Methods

### Participants and procedures

Forty-seven infants participated in the present study. Twenty-four children (12 females) with NDs aged between 3 and 36 months (*M* = 17.88, *SD* = 8.27) were recruited at the Neuropsychiatry and Neurorehabilitation Unit of Scientific Institute IRCCS “*E. Medea*” (Bosisio Parini, Lecco, Italy), where they were hospitalized for assessment and rehabilitation purposes together with their mothers. Inclusion criteria for infants with NDs were: presence of a developmental delay documented by developmental scales (i.e., Griffiths III scales) and/or brain injury outcomes on neurological examination; absence of confirmed genetic syndromes. Among these infants, *n* = 15 (62.5%) have a primary diagnosis of developmental delay and *n* = 9 (37.5%) have a primary diagnosis of cerebral palsy; their mean Developmental Quotient was 64.85 (*SD* = 25.54; range = 20–95).

Twenty-two typically developing children (13 females) aged between 3 and 36 months (*M* = 16.27, *SD* = 9.73) were referred by neonatologists, midwives, paediatricians and nursery school educators from the provinces of Monza-Brianza, Como, Lecco and Milan and recruited using a convenience sampling method. Inclusion criteria for children with TD were: birth at term (≥ 37 weeks of gestation); absence of documented peri- or postnatal pathology. The study was approved by the Ethical Committee of Scientific Institute IRCCS “*E. Medea*” and all the procedures were respectful of the Declaration of Helsinki. All parents signed an informed consent prior to participating in the study. Parents or legal guardians provided informed consent for the study on behalf of their children.

### Measures

#### Infant characteristics

For infants with NDs, demographic and clinical data were extracted from medical records. During their hospital stay, infants with NDs underwent a clinical evaluation which included medical examination, routine screening tests and developmental assessment using the Griffith’s Mental Developmental Scales Third Edition (Griffiths, 1970). For infants with TD, demographic variables and perinatal history were collected using an ad-hoc questionnaire filled out by their mothers. No significant differences were found between the two groups for infant gender (*χ^2^* = 0.38, *p* = 0.54) and age (*t* = 0.61, *p* = 0.55) distribution.

#### DNAm assessment

Buccal swabs for DNAm assessment were collected from all participating infants using DNA Genotek Oragene OC-175. Biological samples were obtained by trained research assistants. DNA was extracted from 0.4 mL saliva aliquots using the manufacturer’s recommended protocol, quantified with Qubit 2.0 (Invitrogen). DNAm levels were determined in DNA using bisulphite modification followed by next generation sequencing. Procedures for DNAm quantification are reported in detail in previous publications from our group (Montirosso et al., 2016; Provenzi et al., 2015). The analysed CpG region of *PIEZO1* (chr16:88846409–88846215) includes 15 CpG (see Table 1 for CpG-specific positions); it is located in intron 1, which exhibits an enhancer-like signature based on ENCODE (Encyclopedia of DNA Elements) data (ENCODE Project Consortium, 2012) and is expected to be involved in the regulation of gene expression. The region was selected because it showed the presence of a differentially methylated site in a previous work (Chu et al., 2018).

**Table 1:** Positions of the selected *PIEZO1* CpG sites human genome assembly GRCh37 (hg19), percentage and range for DNAm level in each CpG sites in the two samples (NDs = neurodevelopmental disorders; TD = typical development).

CpG sites number	Position	Infants with NDs		Infants with TD	
		% DNAm	% DNAm range	% DNAm	% DNAm range
CpG1	chr16:88846409–88846410	31.64	17.9–52.73	43.14	25.38–63.83
CpG2	chr16:88846392–88846393	47.28	29.85–68.56	60.99	41.15–77.82
CpG3	chr16:88846370–88846371	26.38	14.25–38.75	33.72	20.12–47.79
CpG4	chr16:88846329–88846330	76.67	63.74–81.82	78.62	64.45–88.5
CpG5	chr16:88846320–88846321	57.51	37.23–68.97	61.44	41.96–77.44
CpG6	chr16:88846311–88846312	41.62	21.00–58.94	39.69	18.57–51.40
CpG7	chr16:88846292–88846293	65.55	46.65–79.38	69.87	48.39–82.31
CpG8	chr16:88846284–88846285	23.93	15.17–31.91	24.19	5.68–34.01
CpG9	chr16:88846267–88846268	88.41	76.35–93.92	88.37	78.57–93.63
CpG10	chr16:88846244–88846245	92.59	80.22–100	92.41	78.79–96.77
CpG11	chr16:88846234–88846235	57.78	42.89–78.21	53.68	30.8–71.57
CpG12	chr16:88846232–88846233	62.25	31.59–87.55	57.56	39.96–79.19
CpG13	chr16:88846225–88846226	76.60	54.45–86.92	77.13	65.1–89.15
CpG14	chr16:88846217–88846218	52.60	0.16–87.01	60.11	0–92.99
CpG15	chr16:88846214–88846215	52.52	32.46–67.61	51.10	32.21–68.02

All genomic DNA samples were processed and stored following strict security protocols. DNA was extracted from saliva samples and stored under a unique, pseudoanonymized code previously assigned to each participant. This coding system ensured confidentiality; only the principal investigator and specifically authorized researchers had access to the key linking the code to individual identities. Samples were stored at the Molecular Biology Lab of Scientific Institute IRCCS “*E. Medea*” (Bosisio Parini, Lecco, Italy) with badge-controlled access and they were accessible only to designated personnel. Participants were informed of their right to request the destruction of their biological material at any time. To ensure compliance with legal and ethical standards for the protection of genetic data, a Data Protection Impact Assessment (DPIA) was conducted. Biological materials and related data stored for the duration of the project (48 months) would be retained for 25 years, in accordance with analogous national guidelines on clinical trials. Only authorized personnel had permission to re-identify participants via the coded data.

### Data reduction and statistical analyses

The normal distribution of *PIEZO1* DNAm levels at each CpG site was preliminarily checked. To reduce the number of CpG sites to a smaller set of factors which accounted for shared variance (Cecil et al., 2014; Chau et al., 2014) while minimizing information loss, a Principal Component analysis (PCA) was run setting a Varimax rotation and suppressing coefficients lower than 0.30. Parallel analysis (PA) was used to determine the number of components retaining two factors: Principal component1 (PC1) and principal component2 (PC2). Differences in *PIEZO1* DNAm level between PC1 and PC2 between infants with NDs and infants with TD were evaluated using independent sample *t*-test. Statistical analyses were carried out using SPSS29 for Windows, with the significance level set at *p* < 0.05. To adjust for multiple testing, the Benjamini-Hochberg False Discovery Rate (FDR) procedure was applied, with an adjusted *q*-value threshold of 0.012.

## Results

### Principal component analysis

PC1 and PC2 were used for the analyses. PC1 was composed of 7 CpG sites and PC2 was composed of 8 CpG sites accounting for 36.75 and 30.52%, respectively, of the total variance in *PIEZO1* DNAm (Table 2).

**Table 2:** Principal component analysis (PCA) on infants’ *PIEZO1* DNAm conducted among the 15 CpG sites.

*PIEZO1* DNAm sites	Principal components	
	PC1	PC2
CpG1	−0.379	**0.844**
CpG2		0.812
CpG3		0.920
CpG4	0.504	**0.654**
CpG5	0.454	**0.781**
CpG6	0.849	
CpG7		0.804
CpG8	0.744	
CpG9		0.607
CpG10	0.683	
CpG11	0.904	
CpG12	0.921	
CpG13	0.770	
CpG14		0.308
CpG15	0.889	

Loadings on the respective principal components (PCs) are reported in bold. Loadings < 0.30 are not reported.

#### PIEZO1 DNAm level group comparisons

**Figure 1:**
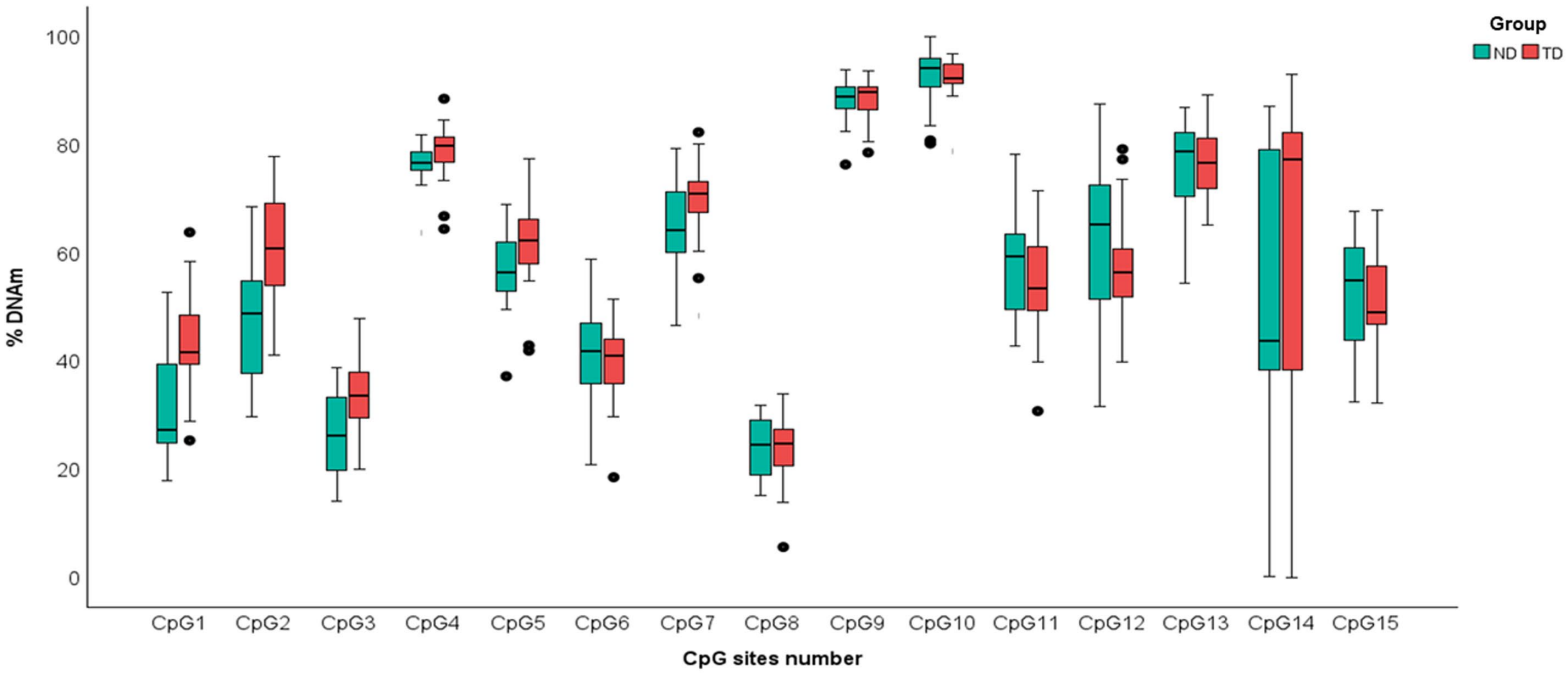
Boxplot of DNAm percentages of each of 15 Cytosine–phosphate–Guanine (CpG) dinucleotides sites of *PIEZO1* in ND and TD groups. Data of infants with NDs are shown in green; data of infants with TD are shown in red.

Significant differences between infants with NDs and infants with TD emerged in PC2. Levels of DNAm in PC2 were significantly lower (*t* = −3.16, *p* = 0.003) in infants with NDs (*M* = 55.75%, *SD* = 6.64) than in infants with TD (*M* = 62.03%, *SD* = 6.84), suggesting hypomethylation in the clinical group. No differences (*t* = 0.818, *p* = 0.209) emerged for PC1 (Figure 1). The results remained consistent after controlling for the infant’s gender and age.

## Discussion

**Figure 2:**
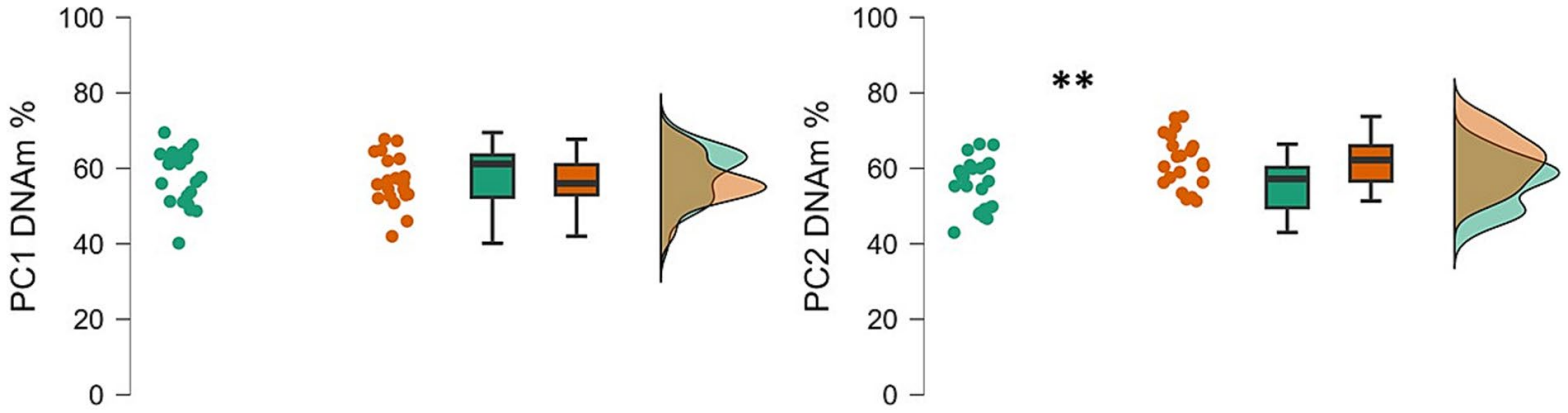
Raincloud plots showing the PC1 and PC2 percentage of DNAm in ND and TD groups. Data of infants with NDs are shown in green; data of infants with TD are shown in red. The plot is made by three component visualizations: individual data values; boxplot of the distribution; density plot that represent the overall shape of the distribution; **p < 0.01.

Neurodevelopmental disorders are associated with dysfunctional brain development, and recent studies suggest that, alongside genetic factors, epigenetic regulation of gene expression may offer novel insight into the molecular mechanisms underlying early brain maturation (Salinas et al., 2020; Trajkova et al., 2024; Khoodoruth et al., 2024). The results of this study provide preliminary evidence of an association between PIEZO1 DNA methylation (DNAm) and NDs in early infancy. Specifically, we observed lower levels of PIEZO1 DNAm in infants with NDs compared to age-matched typically developing children (Figure 2).

From a general perspective, DNA hypomethylation patterns show a relative decrease from typical methylation levels, which have proven to be implicated in several neurological diseases (Li et al., 2024) as well as in neurodevelopmental disorders (Levy et al., 2022; Dall’Aglio et al., 2018). Consistent with this, we may speculate that altered epigenetic regulation of *PIEZO1* may also be involved in neurodevelopment. Although differences between groups were limited to the PC2 component of our DNAm analysis, these findings suggest that *PIEZO1* hypomethylation may be associated with altered neurodevelopmental trajectories, as seen in NDs.

However, in the absence of direct gene expression data, the functional consequences of PIEZO1 hypomethylation remain speculative. While promoter hypomethylation is often linked to increased gene expression, methylation within gene bodies or intergenic regions may have varied or even opposing effects (Zeng et al., 2023). Therefore, we cannot conclusively establish whether the hypomethylation observed in infants with NDs would lead to increased or decreased PIEZO1 expression. If, hypothetically, PIEZO1 hypomethylation results in increased Piezo1 expression, excessive mechanotransduction might influence brain tissue mechanical properties during a critical period of neurodevelopment. Piezo1 overexpression may disrupt the fine-tuned mechanical forces required for developmental processes in the CNS (Chaze et al., 2019; Javier-Torrent et al., 2021). Additionally, recent findings seem to suggest that Piezo1 plays a role in the development and function of the glymphatic and meningeal lymphatic systems, which regulate CNS homeostasis and waste clearance (Zong et al., 2023). In this context, altered PIEZO1 expression, whether increased or decreased, could hypothetically contribute to disrupted fluid dynamics or waste accumulation, as suggested in Alzheimer’s and Parkinson’s disease models (Salvador et al., 2024). Nonetheless, our interpretations remain necessarily cautious, as this study did not assess mRNA or protein expression and cannot establish causality or underlying mechanisms. Future studies integrating methylation and expression data across relevant tissues and developmental stages are needed to better understand the functional role of *PIEZO1* DNAm in neurodevelopment under both healthy and pathological conditions.

This study is not without limitations. First, although *PIEZO1* gene encodes the protein which acts as an ion channel, we did not measure Piezo1-protein expression or gene expression levels (e.g., via mRNA analysis from buccal swabs). As a result, we cannot establish a direct functional link between *PIEZO1* DNAm and protein activity in infants with NDs. While such analyses could offer deeper insight into the biological implications of methylation differences, they were beyond the original aim of the current research project. Future research should integrate epigenetic and expression data to clarify the role of *PIEZO1* in early neurodevelopment as well as in NDs.

Second, only PC2 showed significant differences between groups, while PC1 did not. Given that this is, to the best of our knowledge, the first study to examine *PIEZO1* DNAm in NDs, and one of the very first to explore epigenetic regulation of *PIEZO1* in general, it is too early to hypothesize and attribute specific functional meaning to each component. In the current work, we used the PCA approach to reduce dimensionality, but further research is needed to clarify whether these components reflect biologically distinct regulatory regions or mechanisms and to shed light on the differential biological relevance of these DNAm clusters.

Third, the relatively small sample size limits the generalizability of the results and prevents sample stratification according to specific infant diagnoses. While our ND sample was heterogeneous in terms of diagnosis, such heterogeneity was appropriate for our goals because it can be considered representative of the population of children with ND who are typically referred to neuropsychiatry clinical units.

Fourth, DNAm in buccal cells was used as a proxy for brain methylation. While this is a common approach in neuroepigenetic research in humans, it poses limitations due to possible tissue-specific differences. Braun et al. (2019) reported a satisfying degree of concordance of genome-wide DNAm across commonly used peripheral tissues with DNAm in live human brain tissue, with a good correlation between buccal and brain tissues (r = 0.85) and about 17.4% of CpG sites showing significant within-subject correlations. Moreover, as buccal tissue shares ectodermal origin with brain, it offers a practical and biologically relevant alternative when direct brain access is not feasible. Future studies including also animal models are needed to further clarify this aspect.

Despite these limitations, our study showed that, as in several adult neurodegenerative disease, *PIEZO1* DNAm alteration may serve as a potential epigenetic marker associated with early brain dysfunction, such as that observed in infants with NDs. The bio-molecular key mechanism might be linked to *Piezo1*-protein expression, which could impact the mechanical properties of brain tissue. These preliminary findings suggest that DNAm analysis is a potential powerful methodological tool to understand the underlying mechanisms of dysfunctional brain development, particularly in infants with NDs. Our findings offer promising translational perspectives, suggesting that *PIEZO1* DNAm could serve as an early epigenetic marker of altered brain functioning. This could help identify the potential underlying mechanisms of NDs.

## Data Availability

The datasets presented in this study can be found in online repositories. The names of the repository/repositories and accession number(s) can be found at: https://osf.io/hzakn/.

## References

[ref1] Abuwarda H. Pathak M. M. (2020). Mechanobiology of neural development. Curr. Opin. Cell Biol. 66, 104–111. doi: 10.1016/j.ceb.2020.05.012, PMID: 32687993 PMC7578076

[ref2] Antila S. Karaman S. Nurmi H. Airavaara M. Voutilainen M. H. Mathivet T.. (2017). Development and plasticity of meningeal lymphatic vessels. J. Exp. Med. 214, 3645–3667. doi: 10.1084/jem.20170391, PMID: 29141865 PMC5716035

[ref3] Barnes J. M. Przybyla L. Weaver V. M. (2017). Tissue mechanics regulate brain development, homeostasis and disease. J. Cell Sci. 130, 71–82. doi: 10.1242/jcs.191742, PMID: 28043968 PMC5394781

[ref4] Braun P. R. Han S. Hing B. Nagahama Y. Gaul L. N. Heinzman J. T.. (2019). Genome-wide DNA methylation comparison between live human brain and peripheral tissues within individuals. Transl. Psychiatry 9:47. doi: 10.1038/s41398-019-0376-y, PMID: 30705257 PMC6355837

[ref5] Cecil C. A. Lysenko L. J. Jaffee S. R. Pingault J. B. Smith R. G. Relton C. L.. (2014). Environmental risk, oxytocin receptor gene (OXTR) methylation and youth callous-unemotional traits: a 13-year longitudinal study. Mol. Psychiatry 19, 1071–1077. doi: 10.1038/mp.2014.95, PMID: 25199917 PMC4231290

[ref6] Chau C. M. Ranger M. Sulistyoningrum D. Devlin A. M. Oberlander T. F. Grunau R. E. (2014). Neonatal pain and COMT Val158Met genotype in relation to serotonin transporter (*SLC6A4*) promoter methylation in very preterm children at school age. Front. Behav. Neurosci. 8:409. doi: 10.3389/fnbeh.2014.00409, PMID: 25520635 PMC4251438

[ref7] Chaze C. A. McIlvain G. Smith D. R. Villermaux G. M. Delgorio P. L. Wright H. G.. (2019). Altered brain tissue viscoelasticity in pediatric cerebral palsy measured by magnetic resonance elastography. NeuroImage: Clinical 22:101750. doi: 10.1016/j.nicl.2019.101750, PMID: 30870734 PMC6416970

[ref8] Chen X. Wanggou S. Bodalia A. Zhu M. Dong W. Fan J. J.. (2018). A feedforward mechanism mediated by mechanosensitive ion channel PIEZO1 and tissue mechanics promotes glioma aggression. Neuron 100, 799–815. doi: 10.1016/j.neuron.2018.09.046 30344046

[ref9] Chi S. Cui Y. Wang H. Jiang J. Zhang T. Sun S.. (2022). Astrocytic Piezo1-mediated mechanotransduction determines adult neurogenesis and cognitive functions. Neuron 110, 2984–2999. doi: 10.1016/j.neuron.2022.07.010, PMID: 35963237

[ref10] Choi D. Park E. Choi J. Lu R. Yu J. S. Kim C.. (2024). Piezo1 regulates meningeal lymphatic vessel drainage and alleviates excessive CSF accumulation. Nat. Neurosci. 27, 913–926. doi: 10.1038/s41593-024-01604-8, PMID: 38528202 PMC11088999

[ref11] Chu S. H. Loucks E. B. Kelsey K. T. Gilman S. E. Agha G. Eaton C. B.. (2018). Sex-specific epigenetic mediators between early life social disadvantage and adulthood BMI. Epigenomics 10, 707–722. doi: 10.2217/epi-2017-0146, PMID: 29888956 PMC6367732

[ref12] Coste B. Mathur J. Schmidt M. Earley T. J. Ranade S. Petrus M. J.. (2010). Piezo1 and Piezo2 are essential components of distinct mechanically activated cation channels. Science 330, 55–60. doi: 10.1126/science.1193270, PMID: 20813920 PMC3062430

[ref13] Coste B. Xiao B. Santos J. S. Syeda R. Grandl J. Spencer K. S.. (2012). Piezo proteins are pore-forming subunits of mechanically activated channels. Nature 483, 176–181. doi: 10.1038/nature10812, PMID: 22343900 PMC3297710

[ref14] Coutino B. C. Mayor R. (2021). Reprint of: mechanosensitive ion channels in cell migration. Cells & development 168:203730. doi: 10.1016/j.cdev.2021.203730, PMID: 34456177

[ref15] D'Adamo M. C. Liantonio A. Conte E. Pessia M. Imbrici P. (2020). Ion channels involvement in neurodevelopmental disorders. Neuroscience 440, 337–359. doi: 10.1016/j.neuroscience.2020.05.032, PMID: 32473276

[ref16] Dall’Aglio L. Muka T. Cecil C. A. Bramer W. M. Verbiest M. M. Nano J.. (2018). The role of epigenetic modifications in neurodevelopmental disorders: a systematic review. Neurosci. Biobehav. Rev. 94, 17–30. doi: 10.1016/j.neubiorev.2018.07.011, PMID: 30067938

[ref18] ENCODE Project Consortium (2012). An integrated encyclopedia of DNA elements in the human genome. Nature 489:57. doi: 10.1038/nature11247, PMID: 22955616 PMC3439153

[ref19] Engbers H. M. Nievelstein R. A. J. Gooskens R. H. J. M. Kroes H. Y. Van Empelen R. Braams O.. (2010). The clinical utility of MRI in patients with neurodevelopmental disorders of unknown origin. Eur. J. Neurol. 17, 815–822. doi: 10.1111/j.1468-1331.2009.02927.x, PMID: 20113335

[ref20] Feng J. Fan G. (2009). The role of DNA methylation in the central nervous system and neuropsychiatric disorders. Int. Rev. Neurobiol. 89, 67–84. doi: 10.1016/S0074-7742(09)89004-1, PMID: 19900616

[ref21] Griffiths R. (1970). The abilities of young children: A comprehensive system of mental measurement for the first eight years of life. London: Child Development Research Centre.

[ref22] Gu Y. Gu C. (2014). Physiological and pathological functions of mechanosensitive ion channels. Mol. Neurobiol. 50, 339–347. doi: 10.1007/s12035-014-8654-4, PMID: 24532247 PMC4134430

[ref24] Hemed N. M. Melosh N. A. (2023). An integrated perspective for the diagnosis and therapy of neurodevelopmental disorders–from an engineering point of view. Adv. Drug Deliv. Rev. 194:114723. doi: 10.1016/j.addr.2023.114723, PMID: 36746077

[ref26] Javier-Torrent M. Zimmer-Bensch G. Nguyen L. (2021). Mechanical forces orchestrate brain development. Trends Neurosci. 44, 110–121. doi: 10.1016/j.tins.2020.10.012, PMID: 33203515

[ref28] Khoodoruth M. A. S. Khoodoruth W. N. C. K. Al Alwani R. (2024). Exploring the epigenetic landscape: the role of 5-hydroxymethylcytosine in neurodevelopmental disorders. Cambridge Prisms: Precision Med. 2:e5. doi: 10.1017/pcm.2024.2, PMID: 38699519 PMC11062787

[ref29] Koser D. E. Thompson A. J. Foster S. K. Dwivedy A. Pillai E. K. Sheridan G. K.. (2016). Mechanosensing is critical for axon growth in the developing brain. Nat. Neurosci. 19, 1592–1598. doi: 10.1038/nn.4394, PMID: 27643431 PMC5531257

[ref30] Levy M. A. Relator R. McConkey H. Pranckeviciene E. Kerkhof J. Barat-Houari M.. (2022). Functional correlation of genome-wide DNA methylation profiles in genetic neurodevelopmental disorders. Hum. Mutat. 43, 1609–1628. doi: 10.1002/humu.24446, PMID: 35904121

[ref31] Li L. Chen R. Zhang H. Li J. Huang H. Weng J.. (2024). The epigenetic modification of DNA methylation in neurological diseases. Front. Immunol. 15:1401962. doi: 10.3389/fimmu.2024.1401962, PMID: 39376563 PMC11456496

[ref32] Li Z. Guo W. Zeng T. Yin J. Feng K. Huang T.. (2022). Detecting brain structure-specific methylation signatures and rules for Alzheimer’s disease. Front. Neurosci. 16:895181. doi: 10.3389/fnins.2022.895181, PMID: 35585924 PMC9108872

[ref33] Liu Y. Tian H. Hu Y. Cao Y. Song H. Lan S.. (2022). Mechanosensitive Piezo1 is crucial for periosteal stem cell-mediated fracture healing. Int. J. Biol. Sci. 18:3961. doi: 10.7150/ijbs.71390, PMID: 35844802 PMC9274506

[ref34] Martino F. Perestrelo A. R. Vinarský V. Pagliari S. Forte G. (2018). Cellular mechanotransduction: from tension to function. Front. Physiol. 9:824. doi: 10.3389/fphys.2018.00824, PMID: 30026699 PMC6041413

[ref35] Mastrototaro G. Sessa A. Zaghi M. (2024). “Emerging role of epigenetics in human neurodevelopmental disorders” in Epigenetics in human disease. T. Tollefsbol (editor). *3rd edn* (Academic Press), 285–331. doi: 10.1016/B978-0-443-18661-5.00022-1

[ref36] Momen A. A. Jelodar G. Dehdashti H. (2011). Brain magnetic resonance imaging findings in developmentally delayed children. Int. J. Pediatr. 2011:386984, 1–4. doi: 10.1155/2011/386984, PMID: 22121377 PMC3216390

[ref37] Montirosso R. Provenzi L. Fumagalli M. Sirgiovanni I. Giorda R. Pozzoli U.. (2016). Serotonin transporter gene (SLC6A4) methylation associates with neonatal intensive care unit stay and 3-month-old temperament in preterm infants. Child Dev. 87, 38–48. doi: 10.1111/cdev.12492, PMID: 26822441

[ref38] Morris C. Janssens A. Tomlinson R. Williams J. Logan S. (2013). Towards a definition of neurodisability: a Delphi survey. Dev. Med. Child Neurol. 55, 1103–1108. doi: 10.1111/dmcn.12218, PMID: 23909744

[ref39] Otero-Sobrino Á. Blanco-Carlón P. Navarro-Aguadero M. Á. Gallardo M. Martínez-López J. Velasco-Estévez M. (2023). Mechanosensitive ion channels: their physiological importance and potential key role in cancer. Int. J. Mol. Sci. 24:13710. doi: 10.3390/ijms241813710, PMID: 37762011 PMC10530364

[ref41] Prevarskaya N. Skryma R. Shuba Y. (2018). Ion channels in cancer: are cancer hallmarks oncochannelopathies? Physiol. Rev. 98, 559–621. doi: 10.1152/physrev.00044.2016, PMID: 29412049

[ref43] Provenzi L. Fumagalli M. Sirgiovanni I. Giorda R. Pozzoli U. Morandi F.. (2015). Pain-related stress during the neonatal intensive care unit stay and SLC6A4 methylation in very preterm infants. Front. Behav. Neurosci. 9:99. doi: 10.3389/fnbeh.2015.00099, PMID: 25941480 PMC4403508

[ref46] Radhakrishna U. Vishweswaraiah S. Uppala L. V. Szymanska M. Macknis J. Kumar S.. (2021). Placental DNA methylation profiles in opioid-exposed pregnancies and associations with the neonatal opioid withdrawal syndrome. Genomics 113, 1127–1135. doi: 10.1016/j.ygeno.2021.03.006, PMID: 33711455

[ref47] Ren Y. Huang P. Huang X. Zhang L. Liu L. Xiang W.. (2024). Alterations of DNA methylation profile in peripheral blood of children with simple obesity. Health Inf. Sci. Syst. 12:26. doi: 10.1007/s13755-024-00275-w, PMID: 38505098 PMC10948706

[ref48] Sadikovic B. Aref-Eshghi E. Levy M. A. Rodenhiser D. (2019). DNA methylation signatures in mendelian developmental disorders as a diagnostic bridge between genotype and phenotype. Epigenomics 11, 563–575. doi: 10.2217/epi-2018-0192, PMID: 30875234

[ref49] Salinas R. D. Connolly D. R. Song H. (2020). Invited review: epigenetics in neurodevelopment. Neuropathol. Appl. Neurobiol. 46, 6–27. doi: 10.1111/nan.12608, PMID: 32056273 PMC7174139

[ref50] Salvador A. F. M. Abduljawad N. Kipnis J. (2024). Meningeal lymphatics in central nervous system diseases. Annu. Rev. Neurosci. 47, 323–344. doi: 10.1146/annurev-neuro-113023-103045, PMID: 38648267 PMC12051392

[ref9001] Silbereis J. C. Pochareddy S. Zhu Y. Li M. Sestan N. (2016). The cellular and molecular landscapes of the developing human central nervous system. Neuron 89, 248–268., PMID: 26796689 10.1016/j.neuron.2015.12.008 PMC4959909

[ref51] Trajkova S. Kerkhof J. Sebastiano M. R. Pavinato L. Ferrero E. Giovenino C.. (2024). DNA methylation analysis in patients with neurodevelopmental disorders improves variant interpretation and reveals complexity. Human genetics and genomics. Advances 5:100309. doi: 10.1016/j.xhgg.2024.100309, PMID: 38751117 PMC11216013

[ref52] Velasco-Estevez M. Mampay M. Boutin H. Chaney A. Warn P. Sharp A.. (2018). Infection augments expression of mechanosensing Piezo1 channels in amyloid plaque-reactive astrocytes. Front. Aging Neurosci. 10:332. doi: 10.3389/fnagi.2018.00332, PMID: 30405400 PMC6204357

[ref54] Williams H. J. (2004). Imaging the child with developmental delay. Imaging 16, 174–185. doi: 10.1259/imaging/99057287

[ref55] Zarychanski R. Schulz V. P. Houston B. L. Maksimova Y. Houston D. S. Smith B.. (2012). Mutations in the mechanotransduction protein PIEZO1 are associated with hereditary xerocytosis. Blood 120, 1908–1915. doi: 10.1182/blood-2012-04-422253, PMID: 22529292 PMC3448561

[ref56] Zeng Y. Jain R. Lam M. Ahmed M. Guo H. Xu W.. (2023). DNA methylation modulated genetic variant effect on gene transcriptional regulation. Genome Biol. 24:285. doi: 10.1186/s13059-023-03130-5, PMID: 38066556 PMC10709945

[ref57] Zheng Q. Liu H. Yu W. Dong Y. Zhou L. Deng W.. (2023). Mechanical properties of the brain: focus on the essential role of Piezo1-mediated mechanotransduction in the CNS. Brain Behav. 13:e3136. doi: 10.1002/brb3.3136, PMID: 37366640 PMC10498085

[ref58] Zhou W. Liu X. van Wijnbergen J. W. M. Yuan L. Liu Y. Zhang C.. (2020). Identification of PIEZO1 as a potential prognostic marker in gliomas. Sci. Rep. 10:16121. doi: 10.1038/s41598-020-72886-8, PMID: 32999349 PMC7528027

[ref59] Zong B. Yu F. Zhang X. Pang Y. Zhao W. Sun P.. (2023). Mechanosensitive Piezo1 channel in physiology and pathophysiology of the central nervous system. Ageing Res. Rev. 90:102026. doi: 10.1016/j.arr.2023.102026, PMID: 37532007

